# Metformin Reduces the Risk of Diverticula of Intestine in Taiwanese Patients with Type 2 Diabetes Mellitus

**DOI:** 10.3389/fphar.2021.739141

**Published:** 2021-09-07

**Authors:** Chin-Hsiao Tseng

**Affiliations:** ^1^ Department of Internal Medicine, National Taiwan University College of Medicine, Taipei, Taiwan; ^2^ Division of Endocrinology and Metabolism, Department of Internal Medicine, National Taiwan University Hospital, Taipei, Taiwan; ^3^ Division of Environmental Health and Occupational Medicine of the National Health Research Institutes, Zhunan, Taiwan

**Keywords:** confounding by indication, diverticula of intestine, inverse probability of treatment weighting, metformin, propensity score

## Abstract

**Aim:** To investigate the risk of diverticula of intestine associated with metformin use.

**Methods:** This retrospective cohort study used the Taiwan’s National Health Insurance database to enroll 307,548 ever users and 18,839 never users of metformin. The patients were followed up starting on January 1, 2006 and ending on a date up to December 31, 2011. To address confounding by indication, hazard ratios were derived from Cox regression based on the inverse probability of treatment weighting using propensity score.

**Results:** During follow-up, newly diagnosed cases of diverticula were identified in 1,828 ever users (incidence rate: 125.59 per 100,000 person-years) and 223 never users (incidence rate: 268.17 per 100,000 person-years). Ever users had an approximately 54% lower risk, as shown by the overall hazard ratio of 0.464 (95% confidence interval 0.404–0.534). While patients categorized in each tertile of cumulative duration of metformin therapy were compared to never users, a dose-response pattern was observed with hazard ratios of 0.847 (0.730–0.983), 0.455 (0.391–0.531) and 0.216 (0.183–0.255) for the first (<27.37 months), second (27.37–59.70 months) and third (>59.70 months) tertiles, respectively. The findings were similar when the diagnosis of diverticula was restricted to the small intestine or to the colon. Subgroup analyses suggested that the lower risk of diverticula of intestine associated with metformin use was significant in all age groups of <50, 50–64 and ≥65 years, but the magnitude of risk reduction attenuated with increasing age.

**Conclusion:** Metformin treatment is associated with a significantly reduced risk of diverticula of intestine.

## Introduction

Diverticula of intestine is a common geriatric gastrointestinal disorder in industrialized and urbanized society and its prevalence is higher in the Western countries than in the East (Strate and Morris, 2019). These lesions can either be seen in the small intestine or the colon. Dietary fiber deficiency has been hypothesized as the cause of diverticula because small caliber stools resulting from lack of dietary fiber may cause increased pressure in the intestine leading to herniation of the intestinal mucosa through the muscular layers (Strate and Morris, 2019). The prevalence of diverticula increases with increasing age and more than half of the population older than 60 years of age in the United States have diverticula (Peery et al., 2016). Other risk factors of diverticula include male sex, obesity, dietary patterns (low in fiber and high in red meat, fat and refined grains), physical inactivity, smoking, medications (e.g., non-steroidal anti-inflammatory drugs (NSAIDs), corticosteroid, immunosuppressants and opioid analgesics) and genetic factors (Strate and Morris, 2019; Tursi et al., 2020; Piscopo and Ellul, 2020). Some recent studies suggested a change in the composition of gut microbiota with reduced levels of *Akkermansia* and short-chain fatty acid-producing microbiota in patients with diverticula of intestine (Strate and Morris, 2019; Tursi et al., 2020). Overt inflammation subsequent to obstruction, trauma, ischemia, microperforation and infection of diverticula may cause acute diverticulitis with clinical manifestations of abdominal pain and bleeding (Strate and Morris, 2019; Piscopo and Ellul, 2020). The lifetime risk of developing diverticulitis in patients with diverticula is approximately 10–25% (Hawkins et al., 2020). Complications occur in approximately 12% of the patients with diverticulitis and may include abscess, perforation, peritonitis, stricture, obstruction and/or fistula (Strate and Morris, 2019; Tursi et al., 2020). Antibiotics are always used to treat diverticulitis and surgical resection has been recommended in cases with complicated diverticulitis and recurrence (Strate and Morris, 2019).

Metformin is now the first-line oral antidiabetic drug used for the treatment of hyperglycemia in patients with type 2 diabetes mellitus and more than 150 million diabetes patients are taking metformin over the world (He, 2020). Besides glycemic control, metformin may have multiple pleiotropic benefits including anti-inflammation, anti-microbe, anti-atherosclerosis, anti-neoplasm, anti-aging and immune modulation (Maniar et al., 2017; Chen et al., 2018). In Taiwan, our previous observational studies did suggest that, in comparison to non-users, users of metformin may have a lower risk of colorectal cancer (Tseng, 2012), pulmonary tuberculosis infection (Tseng, 2018a), *Helicobacter pylori* infection (Tseng, 2018b), inflammatory bowel disease (Tseng, 2021a) and hemorrhoids (Tseng, 2021b). All of these support the anti-neoplastic, anti-inflammatory and anti-microbial actions of metformin. After oral intake, metformin distributes to a wide variety of tissues including the gastrointestinal tracts of stomach, small intestine, colon and appendix (Chen et al., 2010).

To our knowledge, there is only one previous study that investigated the potential benefit of metformin on diverticular disease. This is a retrospective case-control study conducted in Australia by Freckelton et al., who compared the use of metformin in 174 diabetes patients with uncomplicated diverticulosis and 175 diabetes patients with acute diverticulitis (Freckelton et al., 2017). They found that patients with acute diverticulitis had a lower rate of metformin use than patients with uncomplicated diverticulosis (44 *versus* 60%, *p* = 0.002) (Freckelton et al., 2017). The investigators concluded that metformin use in diabetes patients with diverticular disease might have a potential benefit in reducing the incidence of acute diverticulitis. However, whether metformin use may prevent the occurrence of diverticula of intestine is an unanswered question. Therefore, we compared the risk of diverticula of intestine in diabetes patients who had been prescribed metformin to those who had never been prescribed metformin.

## Materials and Methods

This is a retrospective cohort study that used the reimbursement database of the National Health Insurance (NHI). Since March 1, 1995, Taiwan has started the implementation of a universal healthcare system, the NHI, that covers >99% of Taiwan’s population. More than 93% of all medical settings and all in-hospitals provide medical care to patients under the coverage of NHI. Computerized medical records including disease diagnoses, drug prescriptions and clinical procedures have to be submitted to the Bureau of the NHI for reimbursement purposes. The reimbursement database of these medical records can be released for academic research if the proposal is approved after institutional review. The Research Ethics Committee of the National Health Research Institutes approved the present study with an approval number of NHIRD-102-175. In accordance to local regulations, informed consent was not required for the use of the database because, for the protection of privacy, personal information has been de-identified before the release of the database.

Throughout the whole study period, the disease coding system used by the NHI was the International Classification of Diseases, Ninth Revision, Clinical Modification (ICD-9-CM). Therefore, ICD-9-CM codes of 250.XX and A-code A181 (A-codes were no longer used after January 1, 2000 in Taiwan) were used to identify patients with a diagnosis of diabetes mellitus. Diverticula of intestine was coded 562 (562.0 for diverticula of small intestine and 562.1 for diverticula of colon).

**FIGURE 1:**
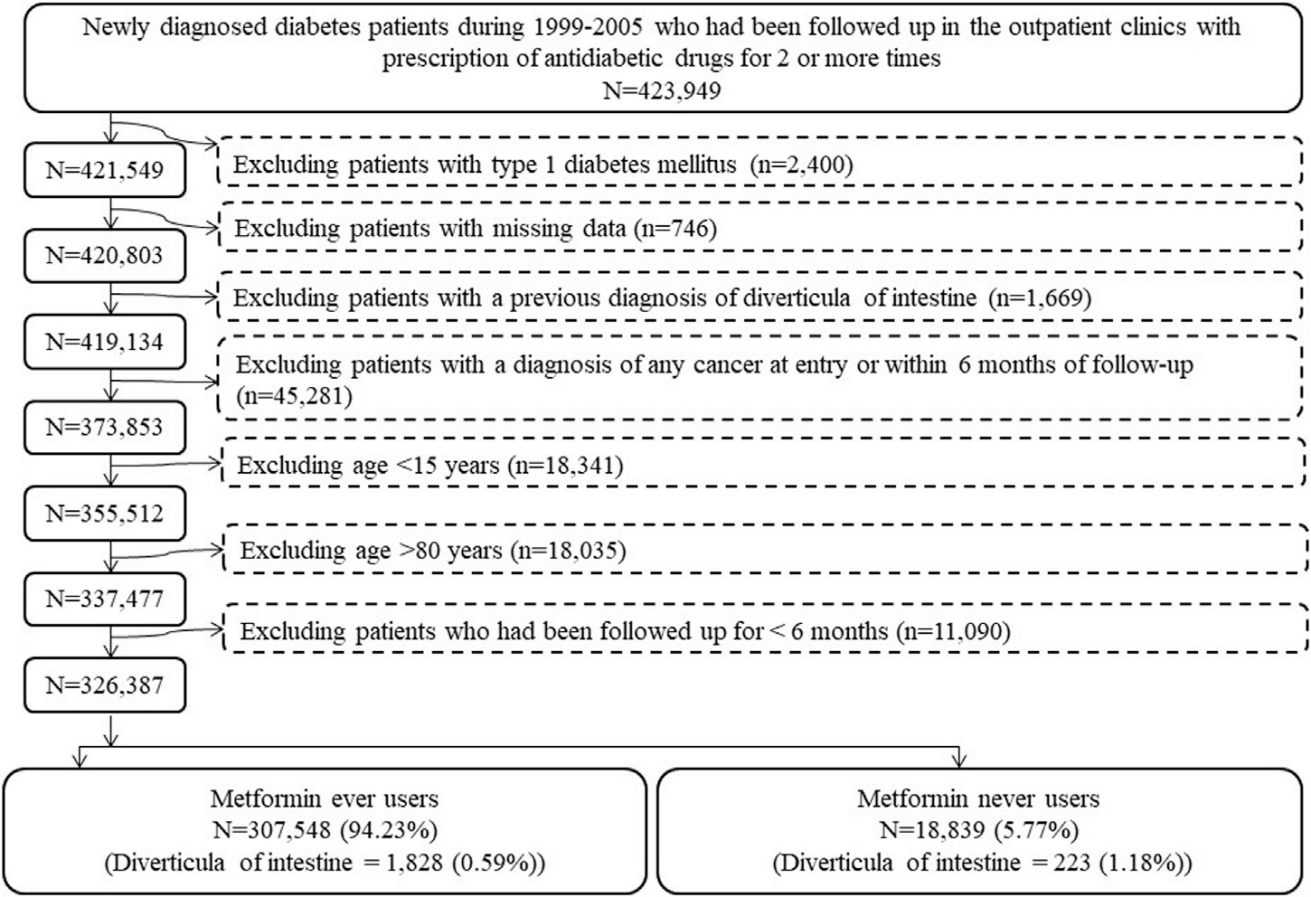
Step-by-step procedures followed to create cohorts of ever users and never users of metformin from the Taiwan’s National Health Insurance database.

The procedures in Figure 1 were followed to create ever users and never users of metformin for analyses. At first, patients with a new diagnosis of diabetes mellitus within the period from 1999 to 2005 and having been prescribed antidiabetic drugs for at least two times in the outpatient clinics were identified (n = 423,949). Ineligible patients were then excluded step-by-step: 1) type 1 diabetes (n = 2,400), 2) missing data (n = 746), 3) diverticula of intestine diagnosed prior to the follow-up starting date (n = 1,669), 4) a diagnosis of any cancer at entry or within 6 months of follow-up (n = 45,281), 5) patients aged <15 years (n = 18,341, these patients were excluded because diverticula of intestine is age-dependent and its occurrence is very rare in children), 6) patients aged >80 years (n = 18,035, these patients were excluded to avoid healthy survivor effect), and 7) patients who had been followed up for <6 months (n = 11,090). As a result, 326,387 patients (307,548 ever users of metformin and 18,839 never users of metformin) were enrolled into the study.

Some variables were retrieved from the database and treated as potential confounders. They were related to basic data, medications used by the patients and disease diagnoses. Basic data included age, sex, occupation and living region. Occupation was divided into four classes according to the Bureau of NHI (I) civil servants, teachers, employees of governmental or private businesses, professionals and technicians (II) people without a specific employer, self-employed people and seamen (III) farmers and fishermen; and (IV) low-income families supported by social welfare and veterans (Tseng, 2019). The living regions of the patients were classified into the following five categories according to the locations of the branch offices of the Bureau of NHI in different geographical regions: Taipei, Northern, Central, Southern, and Kao-Ping/Eastern (Tseng, 2019).

Medications used by the patients were divided into three subgroups: antidiabetic drugs, drugs commonly used by diabetes patients, and drugs that may affect outcome. Antidiabetic drugs included insulin, sulfonylurea, meglitinide, acarbose, rosiglitazone and pioglitazone. Drugs commonly used by diabetes patients included angiotensin-converting enzyme inhibitor and/or angiotensin receptor blocker, calcium channel blocker, statin, fibrate and aspirin. Drugs that may affect the outcome of acute diverticulitis included NSAIDs (continuous use for ≥90 days and not including aspirin), immunosuppressants (continuous use ≥90 days and included corticosteroids, calcineurin inhibitors and/or inosine-5′-monophosphate dehydrogenase inhibitors) (Tseng, 2019), opioid analgesics [alfentanil, buprenorphine, codeine, fentanyl, hydromorphone, morphine, nalbuphine, oxycodone, pethidine, propoxyphene and tramadol (Chen et al., 2021)] and selective serotonin re-uptake inhibitor. NSAIDs and immunosuppressants are commonly used in clinical practice. We assumed that a short period of their use would probably not affect the risk of diverticula of intestine and therefore defined users only among those who continuously used these drugs for ≥90 days.

Disease diagnoses were divided into the following three categories: major comorbidities of diabetes, diabetes-related complications, and common comorbidities that may affect the exposure/outcome. These disease diagnoses were selected because they might have a potential correlation with either the exposure or the outcome or because they might affect the patients’ life expectancy, leading to a shortened follow-up duration and a biased estimate of person-years in the calculation of incidence. Disease diagnoses that might require the long-term use of antibiotics, corticosteroid and anti-inflammatory drugs were especially considered because these drugs might have an impact on the risk of diverticula of intestine. Major comorbidities of diabetes included hypertension, dyslipidemia and obesity. Diabetes-related complications included nephropathy, eye diseases, diabetic polyneuropathy, stroke, ischemic heart disease and peripheral arterial disease. Common comorbidities that may affect the exposure/outcome included chronic obstructive pulmonary disease (a surrogate for smoking), tobacco abuse, alcohol-related diagnoses, heart failure, Parkinson’s disease, dementia, head injury, valvular heart disease, gingival and periodontal diseases, pneumonia, osteoporosis, arthropathies and related disorders, psoriasis and similar disorders, dorsopathies, liver cirrhosis, other chronic non-alcoholic liver diseases, hepatitis B virus infection, hepatitis C virus infection, human immunodeficiency virus infection, organ transplantation, *Helicobacter pylori* infection (041.86), peptic ulcer site unspecified (533), appendicitis (540), noninfective enteritis and colitis (555–558), irritable bowel syndrome (564.1), anal fissure/fistula (565), abscess of anal/rectal regions (566), episodic mood disorders (296), depressive disorder (311) and drug dependence (304). The ICD-9-CM codes for the last ten disease diagnoses are shown in parentheses and the codes for the others can be seen in a previous paper (Tseng, 2021a).

Some previous studies have assessed the accuracy of the ICD-9-CM codes labeled in the NHI database for various disease diagnoses (Chang, 2004; Hsieh et al., 2019). The codes of 250.XX used for the diagnosis of diabetes mellitus have a sensitivity of 90.9% and a positive predictive value of 90.2% (Hsieh et al., 2019). In another study, moderate to substantial Kappa values ranging from 0.55 to 0.86 were noted for the agreements between claim data and medical records (Chang, 2004).

Standardized difference was calculated according to Austin and Stuart for each variable (Austin and Stuart, 2015). Potential confounding from a variable was considered if its value of standardized difference was >10%.

The dose-response effect was assessed by analyzing the tertiles of months of metformin exposure calculated as the cumulative duration of metformin therapy. Incidence density was calculated with regards to metformin exposure, in never users, ever users, and users categorized by the tertiles of cumulative duration. Follow-up was set to start on January 1, 2006. New cases of diverticula identified after follow-up starting date were the incidence numerator. The person-years of follow-up was the incidence denominator, calculated since the starting date of follow-up until whichever of the following events occurred first, up to the date of December 31, 2011: a new diagnosis of diverticula of intestine, death or the last available record in the reimbursement database.

Cumulative incidence functions for diverticula of intestine were plotted with regards to metformin exposure. We first plotted the curves for ever and never users, and then the curves for users categorized in each tertile of cumulative duration and never users were plotted. The differences with regards to metformin exposure were tested by Gray’s test.

Logistic regression was then used to create propensity scores from all variables in Table 1 plus the date of entry. Hazard ratios were derived from Cox proportional hazards regression using the method of inverse probability of treatment weighting (IPTW) based on propensity scores, as recommended by Austin to reduce confounding by indication (Austin, 2013). In the main analyses, the overall hazard ratio was estimated by comparing ever users to never users; and for the evaluation of a dose-response relationship, the hazard ratio comparing each tertile of cumulative duration of metformin therapy to never users was estimated. To further examine whether the effects could be consistent for diverticula of small intestine and for diverticula of colon, hazard ratios were also estimated for the respective locations of diverticula (sub-location analyses).

**TABLE 1:** Comparison of characteristics in study subjects by metformin exposure.

Variables	Never users of metformin (n = 18,839)		Ever users of metformin (n = 307,548)		Standardized difference
	N	%	n	%	
**Basic data**					
Age^a^ (years)	61.58	11.75	57.86	11.40	−34.15
Sex (men)	10,556	56.03	164,145	53.37	−5.48
** Occupation**					
I	7,061	37.48	121,114	39.38	
II	3,448	18.30	67,034	21.80	9.07
III	4,277	22.70	63,635	20.69	−4.89
IV	4,053	21.51	55,765	18.13	−9.09
** Living region**					
Taipei	6,312	33.50	103,209	33.56	
Northern	1,962	10.41	37,148	12.08	5.48
Central	3,291	17.47	54,846	17.83	0.92
Southern	3,269	17.35	49,827	16.20	−3.12
Kao-Ping and Eastern	4,005	21.26	62,518	20.33	−2.01
**Major comorbidities of diabetes**					
Hypertension	14,340	76.12	215,572	70.09	−14.80
Dyslipidemia	11,356	60.28	209,729	68.19	17.56
Obesity	392	2.08	13,518	4.40	13.24
**Diabetes-related complications**					
Nephropathy	4,990	26.49	50,854	16.54	−27.76
Eye diseases	1,730	9.18	44,665	14.52	17.03
Diabetic polyneuropathy	1,924	10.21	50,670	16.48	19.12
Stroke	5,228	27.75	63,171	20.54	−18.78
Ischemic heart disease	7,803	41.42	109,136	35.49	−13.60
Peripheral arterial disease	3,107	16.49	50,820	16.52	−0.49
**Antidiabetic drugs**					
Insulin	1,555	8.25	6,442	2.09	−30.80
Sulfonylurea	13,660	72.51	200,643	65.24	−11.07
Meglitinide	1,597	8.48	11,106	3.61	−22.58
Acarbose	2,117	11.24	15,295	4.97	−22.70
Rosiglitazone	548	2.91	13,471	4.38	8.46
Pioglitazone	461	2.45	7,279	2.37	0.35
**Drugs commonly used by diabetes patients**					
Angiotensin converting enzyme inhibitor and/or angiotensin receptor blocker	11,604	61.60	178,618	58.08	−8.19
Calcium channel blocker	11,034	58.57	155,877	50.68	−17.30
Statins	7,456	39.58	135,583	44.09	9.59
Fibrates	5,146	27.32	98,184	31.92	10.49
Aspirin	9,434	50.08	146,772	47.72	−5.68
**Drugs that may affect the outcome**					
Non-steroidal anti-inflammatory drugs	6,672	35.42	92,708	30.14	−12.51
Selective serotonin re-uptake inhibitors	1,492	7.92	20,197	6.57	−6.27
Opioid analgesics	3,221	17.10	49,058	15.95	−4.44
Immunosuppressants	983	5.22	9,771	3.18	−12.45
**Common comorbidities that may affect the exposure/outcome**					
Chronic obstructive pulmonary disease	8,062	42.79	122,328	39.78	−7.44
Tobacco abuse	283	1.50	6,069	1.97	3.85
Alcohol-related diagnoses	1,071	5.69	15,701	5.11	−3.20
Heart failure	3,296	17.50	35,578	11.57	−19.32
Parkinson’s disease	539	2.86	4,765	1.55	−10.18
Dementia	1,130	6.00	12,293	4.00	−10.85
Head injury	234	1.24	3,482	1.13	−1.33
Valvular heart disease	1,846	9.80	20,735	6.74	−12.73
Gingival and periodontal diseases	14,233	75.55	244,398	79.47	9.73
Pneumonia	2,120	11.25	24,817	8.07	−13.23
Osteoporosis	3,717	19.73	51,437	16.72	−8.78
Arthropathies and related disorders	12,972	68.86	208,104	67.67	−3.10
Psoriasis and similar disorders	387	2.05	6,818	2.22	1.01
Dorsopathies	12,812	68.01	213,271	69.35	2.72
Liver cirrhosis	962	5.11	9,466	3.08	−11.60
Other chronic non-alcoholic liver diseases	1,436	7.62	26,587	8.64	3.83
Hepatitis B virus infection	347	1.84	4,766	1.55	−3.01
Hepatitis C virus infection	814	4.32	9,963	3.24	−6.50
Human immunodeficiency virus infection	14	0.07	157	0.05	−1.12
Organ transplantation	115	0.61	427	0.14	−10.04
*Helicobacter pylori* infection	109	0.58	1,554	0.51	−1.12
Peptic ulcer site unspecified	7,064	37.50	106,875	34.75	−6.75
Appendicitis	283	1.50	4,854	1.58	0.41
Noninfective enteritis and colitis	8,723	46.30	146,646	47.68	2.60
Irritable bowel syndrome	2,485	13.19	37,527	12.20	−3.61
Anal fissure/fistula	411	2.18	7,304	2.37	1.14
Abscess of anal/rectal regions	256	1.36	5,298	1.72	3.12
Episodic mood disorders	934	4.96	13,633	4.43	−3.21
Depressive disorder	547	2.90	8,278	2.69	−1.84
Drug dependence	88	0.47	923	0.30	−3.04

a Age is expressed as mean and standard deviation.

See “*Materials and Methods*” section for categories of occupation.

Sensitivity analyses that estimated the hazard ratios comparing ever users to never users were conducted in more restricted subgroups as follows: I. Patients were censored when 4 months have elapsed from the date of the last prescription; II. Patients receiving other antidiabetic drugs before metformin initiation were excluded (This excluded the possibility of carry-over effect that might have been incurred by other antidiabetic drugs prescribed before metformin initiation.); III. Patients who had been followed up for less than 12 months were excluded; IV. Patients who had a metformin treatment duration less than 12 months were excluded; V. Analysis restricted to patients enrolled from 1999 to 2002; VI. Analysis restricted to patients enrolled from 2003 to 2005; VII. Patients receiving two consecutive prescriptions of metformin that spanned more than 4 months were excluded (The NHI does not allow any prescription of drugs for a period of longer than 3 months. Therefore, these patients had a delayed drug refill of metformin and might have represented those not regularly followed up.); VIII. Patients having been treated with incretin-based therapies during follow-up were excluded [Use of dipeptidyl peptidase 4 inhibitors may lead to changes in the composition of gut microbiota (Ryan et al., 2020) and incretin-based therapy was not reimbursed by the NHI before 2009 in Taiwan. These patients were excluded so that the potential impact of incretin-based therapies that happened to be prescribed during follow-up could be avoided.]; IX. Diverticula of intestine defined as a primary diagnosis made at hospitalization (These patients might represent those with acute diverticulitis and the diagnosis might have been supported by laboratory examination done during hospitalization); X. Patients aged <50 years were included; XI. Patients aged 50–64 years were included; XII. Patients aged ≥65 years were included; XIII. Analysis restricted to male patients; and XIV. Analysis restricted to female patients; XV. Patients who received metformin treatment for <90 defined daily dose per year were excluded. Patients aged <15 years and >80 years were excluded in all the previous analyses (Figure 1). To examine whether the findings could be consistent if these patients were not excluded, an additional sensitivity analysis was conducted by included patients of all ages (Model XVI).

Version 9.4 of SAS (SAS Institute, Cary, NC) was the software used for the performance of statistical analyses. Statistical significance was indicated by a *p* value <0.05.

## Results

Table 1 compares the characteristics in the study subjects divided as never users and ever users of metformin. Standardized difference >10% was observed for age, hypertension, dyslipidemia, obesity, nephropathy, eye diseases, diabetic polyneuropathy, stroke, ischemic heart disease, insulin, sulfonylurea, meglitinide, acarbose, calcium channel blocker, fibrate, NSAIDs, immunosuppressants, heart failure, Parkinson’s disease, dementia, valvular heart disease, pneumonia, liver cirrhosis and organ transplantation. Because significant differences in some potential confounders could be seen between ever users and never users of metformin, this justified the use of the IPTW method to estimate hazard ratios recommended by Austin (Austin, 2013).

**FIGURE 2:**
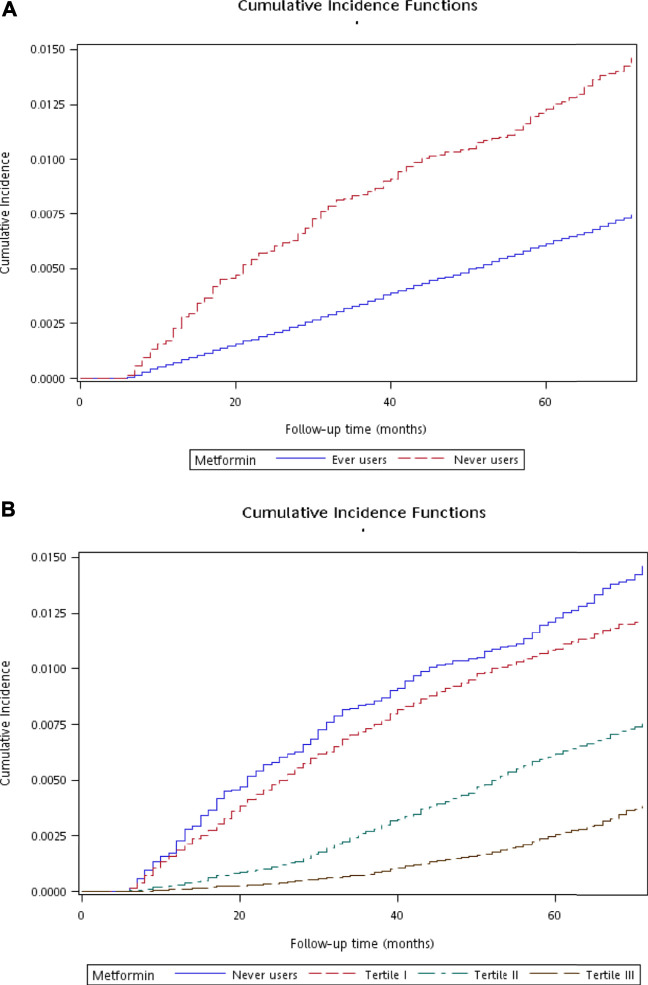
Plots showing the cumulative incidence function of diverticula of intestine. **(A)** compares ever users to never users (*p* < 0.01 by Gray’s test) and **(B)** compares ever users in the three tertiles of cumulative duration of metformin therapy to never users (*p* < 0.01 by Gray’s test).

Figure 2 shows the plots of cumulative incidence function of diverticula of intestine with regards to metformin exposure. The curves for never users and ever users are shown in Figure 2A, which indicates a lower cumulative incidence in ever users in comparison to never users (*p* < 0.01 derived from Gray’s test). The curves for never users and for each tertile of cumulative duration are shown in Figure 2B. The cumulative incidence reduced with regards to increasing cumulative dose of metformin exposure (*p* < 0.01 derived from Gray’s test).

Table 2 shows the incidence of diverticula in different subgroups of metformin exposure and the hazard ratios comparing subgroups of metformin exposure to never users in the main analyses for diverticula of intestine (in small intestine or colon) and in the sub-location analyses for diverticula of small intestine and diverticula of colon, respectively. In the main analyses, the incidence rate of diverticula of intestine was 268.17 per 100,000 person-years in never users after a median follow-up duration of 5.4 years and was 125.59 per 100,000 person-years in ever users after a median follow-up of 5.6 years. A significantly 54% lower risk in ever users was indicated by the overall hazard ratio and a dose-response relationship was noted in the tertile analyses. The findings in the sub-location analyses were very similar to the main analyses.

**TABLE 2:** Incidences of diverticula in different subgroups of metformin exposure and hazard ratios comparing subgroups of metformin exposure to never users of metformin.

Metformin use	Incident case number	Cases followed	Person-years	Incidence rate (per 100,000 person-years)	Hazard ratio	95% confidence interval	*p* value
**Main analyses**							
** Diverticula of intestine**							
Never users	223	18,839	83,154.93	268.17	1.000		
Ever users	1,828	307,548	1,455,522.08	125.59	0.464	(0.404–0.534)	<0.0001
** Tertiles of cumulative duration of metformin therapy (months)**							
Never users	223	18,839	83,154.93	268.17	1.000		
<27.37	829	101,552	358,867.52	231.00	0.847	(0.730–0.983)	0.0288
27.37–59.70	621	101,386	499,252.24	124.39	0.455	(0.391–0.531)	<0.0001
>59.70	378	104,610	597,402.32	63.27	0.216	(0.183–0.255)	<0.0001
**Sub-location analyses**							
** Diverticula of small intestine**							
Never users	25	18,839	83,633.48	29.89	1.000		
Ever users	228	307,548	1,458,937.05	15.63	0.519	(0.343–0.784)	0.0019
** Tertiles of cumulative duration of metformin therapy (months)**							
Never users	25	18,839	83,633.48	29.89	1.000		
<26.93	94	101,552	360,615.71	26.07	0.854	(0.548–1.332)	0.4873
26.93–59.23	80	101,386	500,394.29	15.99	0.533	(0.340–0.836)	0.0061
>59.23	54	104,610	597,927.06	9.03	0.285	(0.177–0.458)	<0.0001
** Diverticula of colon**							
Never users	202	18,839	83,227.94	242.71	1.000		
Ever users	1,631	307,548	1,455,941.78	112.02	0.458	(0.395–0.530)	<0.0001
** Tertiles of cumulative duration of metformin therapy (months)**							
Never users	202	18,839	83,227.94	242.71	1.000		
<26.93	749	101,552	359,058.88	208.60	0.846	(0.723–0.989)	0.0357
26.93–59.23	552	101,386	499,391.05	110.53	0.446	(0.380–0.524)	<0.0001
>59.23	330	104,610	597,491.85	55.23	0.207	(0.174–0.247)	<0.0001

The sensitivity analyses consistently supported a lower risk of diverticula of intestine in patients who had been treated with metformin (Table 3). The significant risk reduction of diverticula of intestine in ever users could be demonstrated in all three age subgroups of <50 years, 50–64 years and ≥65 years, although the magnitude of risk reduction attenuated with increasing age. The preventive effect of metformin could be similar shown in men and in women.

**TABLE 3:** Sensitivity analyses.

Metformin use	Incident case number	Cases followed	Person-years	Incidence rate (per 100,000 person-years)	Hazard ratio	95% confidence interval	*p* value
**I. Patients were censored when 4 months have elapsed from the date of the last prescription**							
Never users	223	18,839	83,154.93	268.17	1.000		
Ever users	1,520	307,548	1,256,724.90	120.95	0.454	(0.394–0.522)	<0.0001
**II. Patients receiving other antidiabetic drugs before metformin initiation were excluded**							
Never users	223	18,839	83,154.93	268.17	1.000		
Ever users	883	143,194	686,610.28	128.60	0.475	(0.410–0.550)	<0.0001
**III. Patients who had been followed up for less than 12 months were excluded**							
Never users	186	17,799	82,377.18	225.79	1.000		
Ever users	1,620	299,285	1,449,322.43	111.78	0.489	(0.420–0.569)	<0.0001
**IV. Patients who had a metformin treatment duration less than 12 months were excluded**							
Never users	223	18,839	83,154.93	268.17	1.000		
Ever users	1,454	261,533	1,311,843.09	110.84	0.403	(0.350–0.465)	<0.0001
**V. Analysis restricted to patients enrolled from 1999 to 2002**							
Never users	90	8,238	35,508.07	253.46	1.000		
Ever users	1,043	168,187	810,208.96	128.73	0.499	(0.403–0.619)	<0.0001
**VI. Analysis restricted to patients enrolled from 2003 to 2005**							
Never users	133	10,601	47,646.86	279.14	1.000		
Ever users	785	139,361	645,313.13	121.65	0.434	(0.361–0.522)	<0.0001
**VII. Patients receiving two consecutive prescriptions of metformin that spanned more than 4 months were excluded**							
Never users	223	18,839	83,154.93	268.17	1.000		
Ever users	494	92,455	415,366.03	118.93	0.442	(0.378–0.518)	<0.0001
**VIII. Patients having been treated with incretin-based therapies during follow-up were excluded**							
Never users	219	17,732	77,964.60	280.90	1.000		
Ever users	1,696	240,053	1,109,450.64	152.87	0.541	(0.470–0.623)	<0.0001
**IX. Diverticula of intestine defined as a primary diagnosis made at hospitalization**							
Never users	55	18,839	83,600.34	65.79	1.000		
Ever users	483	307,548	1,458,361.61	33.12	0.500	(0.378–0.661)	<0.0001
**X. Patients aged < 50 years were included**							
Never users	28	3,219	14,810.81	189.05	1.000		
Ever users	289	74,719	366,663.02	78.82	0.414	(0.281–0.610)	<0.0001
**XI. Patients aged 50–64 years were included**							
Never users	82	7,377	33,397.33	245.53	1.000		
Ever users	804	143,108	684,570.26	117.45	0.475	(0.378–0.596)	<0.0001
**XII. Patients aged ≥ 65 years were included**							
Never users	113	8,243	34,946.79	323.35	1.000		
Ever users	735	89,721	404,288.80	181.80	0.559	(0.458–0.681)	<0.0001
**XIII. Analysis restricted to male patients**							
Never users	122	10,556	46,390.12	262.99	1.000		
Ever users	967	164,145	769,988.80	125.59	0.474	(0.392–0.572)	<0.0001
**XIV. Analysis restricted to female patients**							
Never users	101	8,283	36,764.80	274.72	1.000		
Ever users	861	143,403	685,533.28	125.60	0.453	(0.369–0.557)	<0.0001
**XV. Patients who received metformin treatment for <90 defined daily dose per year were excluded**							
Never users	223	18,839	83,154.93	268.17	1.000		
Ever users	1,763	298,215	1,420,998.97	124.07	0.458	(0.399–0.527)	<0.0001
**XVI. Including patients of all ages**							
Never users	265	21,546	92,274.23	287.19	1.000		
Ever users	1,972	321,255	1,505,716.47	130.97	0.451	(0.397–0.513)	<0.0001

## Discussion

This observational study first showed a preventive role of metformin use in the occurrence of diverticula of intestine in patients with type 2 diabetes mellitus. The results were consistent in the main analyses and the sub-location analyses (Table 2) and in the sensitivity analyses (Table 3). The dose-response pattern in the tertile analyses (Table 2) suggested a potential cause-effect relationship.

Although not yet researched, the anti-inflammatory, anti-microbial and immune modulatory properties of metformin (Maniar et al., 2017; Chen et al., 2018) might have contributed to such a reduced risk. Gut microbiota play important role in the integrity of gut barrier function (Régnier et al., 2021), and gut immune-microbe interaction affects the development of diverticula of intestine (Strate and Morris, 2019; Tursi et al., 2020). *Akkermansia muciniphila* (a mucin-degrading bacterium that colonizes in the mucus layer and improves intestinal barrier function) and microbiota that can produce short-chain fatty acids such as acetate, propionate and butyrate from dietary fiber are reduced in patients with diverticula of intestine (Strate and Morris, 2019; Tursi et al., 2020). It is interesting that metformin may induce the proliferation of *Akkermansia muciniphila* and short-chain fatty acid-producing microbiota in the intestine (Ryan et al., 2020; Lee et al., 2021; de la Cuesta-Zuluaga et al., 2017). As a result, metformin may modulate immune response, inhibit inflammation, reduce permeability and improve tight junction in the gut (Lee et al., 2021). In recent years, bile acids have been shown to play an important role in the regulation of intestinal functions (Yang et al., 2021). It is interesting that metformin may inhibit the reabsorption of bile acids leading to increased availability of bile acids to the gut (Lee et al., 2021).

This study may have several clinical implications. First, the prevention of metformin on diverticula of intestine supports an additional bonus besides other pleiotropic effects by using metformin for the treatment of type 2 diabetes mellitus. The clinical and economical burdens of diverticula of intestine can be anticipated to reduce by the use of a very inexpensive antidiabetic drug. Second, because of the dose-response effect in terms of cumulative duration of metformin use (Table 2) and the potential mechanisms independent of glycemic control, the continuous use of metformin in patients without any contraindication is recommended when other antidiabetic drugs are required for further improvement of hyperglycemia. Third, because the use of the anti-inflammatory drug mesalamine (mesalazine or 5-aminosalicylic acid) for the prevention of recurrent diverticulitis has not been successful in a meta-analysis that included six trials with 2,461 patients (Strate and Morris, 2019; Khan et al., 2018), the findings of the present study together with the findings of the previous study conducted by Freckelton et al. (Freckelton et al., 2017) support the potential usefulness of metformin in the prevention of the occurrence of diverticular disease and the progression of the disease into acute diverticulitis. These findings in observational studies give sufficient rationale for the conduction of clinical trials to confirm and prove such beneficial effects of metformin.

The present study may have some limitations. First, measurement data of potential confounders, biochemical profiles, inflammatory biomarkers, gut microbiota and genetic factors were not available in the NHI database and only diagnostic codes could be used as surrogates for adjustment. Second, although we tried to avoid confounding by indication by balancing the baseline characteristics between ever users and never users of metformin by applying the IPTW method using propensity scores, it was not sure whether residual confounding could still remain. Third, it was recognized that the role of unmeasured confounders could never be assessed and their effects would not be adjusted for by statistical methods. Fourth, misclassification of disease diagnoses could not be excluded in the database. However, the misclassification was expected to be nondifferential, which might have only biased the estimated hazard ratios toward the null. The consistency of the findings in different analyses (Tables 2 and 3) suggested the robustness of a preventive role of metformin. Fifth, for decision making and clinical application, knowledge of absolute risk reduction and number needed to treat is important (Ranganathan et al., 2016). As the incidence of diverticula of intestine was low, the absolute risk reduction calculated was too small (223/18839 - 1828/307,548 = 0.59%) and the number needed to treat (the reciprocal of absolute risk reduction) of 170 seemed to be too large as to be cost-effective to use metformin for the prevention of diverticula of intestine, especially in non-diabetes people.

Although Austin recommended the IPTW method among others that used the propensity score (Austin, 2013), to further assure that the finding of a preventive role of metformin on diverticula of intestine was robust, we had conducted additional models by using the traditional Cox regression after adjustment for all covariates and after adjustment for propensity score. The findings were very similar and did not affect the conclusion of the study (data not shown). Furthermore, we analyzed the database in a cross-sectional manner at the end of the enrollment period and at the end of the follow-up, respectively. We found that the respective odds ratios for metformin use *versus* non-use were 0.538 (0.465–0.623) and 0.528 (0.456–0.612); and the odds ratios for the respective tertiles of cumulative duration of metformin therapy were 1.114 (0.950–1.305), 0.553 (0.471–0.648) and 0.251 (0.211–0.298) when analyzed at the end of the enrollment period and were 1.083 (0.924–1.270), 0.537 (0.458–0.631) and 0.248 (0.208–0.294) when analyzed at the end of follow-up. These additional analyses suggested that the conclusion would not be affected by either analyzing the database in a prospective cohort or in a cross-sectional cohort. When the cumulative incidence functions with Gray’s test in Figure 2 were reanalyzed by the corresponding Kaplan-Meier curves with logrank test, the *p* values were <0.01 in both the analyses for ever *versus* never users and for ever users in the three tertiles of cumulative duration of metformin therapy *versus* never users. Therefore, all additional analyses suggested the robustness of a preventive role of metformin on diverticula of intestine in a dose-response pattern.

This study has several strengths related to the use of a large population-based database and the design of the study. First, selection bias and lack of statistical power could be avoided because of the high coverage rate of the nationwide NHI, the enrollment of a large sample size of all diabetes patients during a long period of time (1999–2005) and the long follow-up duration (from 2006–2011). Therefore, the findings could be more safely generalized to the whole population. Second, by using preexisting medical records, recall bias and self-reporting bias could be prevented. Third, prevalent user bias could be reduced because we included only patients who were newly diagnosed of diabetes mellitus and were new users of metformin. Fourth, immortal time bias may result from inappropriate assignment of treatment status and/or miscalculation of follow-up time. In the present study we included only patients with a definite diagnosis of diabetes mellitus by restricting the patients to those who had received at least two times of prescription of antidiabetic drugs (Figure 1). Because longitudinal information of drug prescription was obtained from the NHI database, misclassification of metformin treatment status was not likely and the cumulative doses could be calculated with less bias. In the calculation of follow-up person-years we deliberately excluded the immortal time that might happen between diabetes diagnosis and the initiation of antidiabetic drugs and the immortal time when the outcome was less likely to occur during the initial follow-up period of <6 months. It should also be pointed out that the immortal time between the date of hospital discharge and the date when discharged drugs are refilled would not be a problem in Taiwan because all drugs prescribed at the time of hospital discharge can be readily obtained on the same day. Fifth, different socioeconomic status can be an important factor associated with detection bias in some countries but this would not cause much problem in this study because the cost-sharing is very low in our healthcare system. Furthermore, much expense can actually be waived in some groups of patients (e.g., patients with low income, veterans and patients refilling prescriptions for chronic diseases).

In summary, this is the first study that shows a preventive role of metformin in diverticula of intestine. Although this benefit can be observed in all age groups, the risk reduction attenuates with increasing age. Because observational study design may have some inherent limitations, the findings should be confirmed by additional studies and better by clinical trials. The cost-effectiveness of metformin use in the prevention of diverticula of intestine is not known, especially in people without diabetes. However, because of the multiple benefits of metformin beyond glycemic control, it should be appropriate to recommend metformin as the first-line antidiabetic drug.

## Data Availability

The datasets presented in this article are not readily available because public availability of the dataset is restricted by local regulations to protect privacy. Requests to access the datasets should be directed to ccktsh@ms6.hinet.net.
